# The level of activity of the alternative lengthening of telomeres correlates with patient age in IDH-mutant ATRX-loss-of-expression anaplastic astrocytomas

**DOI:** 10.1186/s40478-019-0833-0

**Published:** 2019-11-09

**Authors:** Nathalie Grandin, Bruno Pereira, Camille Cohen, Pauline Billard, Caroline Dehais, Catherine Carpentier, Ahmed Idbaih, Franck Bielle, François Ducray, Dominique Figarella-Branger, Jean-Yves Delattre, Marc Sanson, Patrick Lomonte, Delphine Poncet, Pierre Verrelle, Michel Charbonneau, POLA network, C. Desenclos, H. Sevestre, P. Menei, A. Rousseau, T. Cruel, S. Lopez, M-I Mihai, A. Petit, C. Adam, F. Parker, P. Dam-Hieu, I. Quintin-Roué, S. Eimer, H. Loiseau, L. Bekaert, F. Chapon, D. Ricard, C. Godfraind, T. Khalil, D. Cazals-Hatem, T. Faillot, C. Gaultier, M. C. Tortel, I. Carpiuc, P. Richard, W. Lahiani, H. Aubriot-Lorton, F. Ghiringhelli, C. A. Maurage, C. Ramirez, E. M. Gueye, F. Labrousse, O. Chinot, L. Bauchet, V. Rigau, P. Beauchesne, G. Gauchotte, M. Campone, D. Loussouarn, D. Fontaine, F. Vandenbos-Burel, A. Le Floch, P. Roger, C. Blechet, M. Fesneau, A. Carpentier, J. Y. Delattre, S. Elouadhani-Hamdi, M. Polivka, D. Larrieu-Ciron, S. Milin, P. Colin, M. D. Diebold, D. Chiforeanu, E. Vauleon, O. Langlois, A. Laquerriere, F. Forest, M. J. Motso-Fotso, M. Andraud, G. Runavot, B. Lhermitte, G. Noel, S. Gaillard, C. Villa, N. Desse, C. Rousselot-Denis, I. Zemmoura, E. Cohen-Moyal, E. Uro-Coste, F. Dhermain

**Affiliations:** 1 Laboratoire GReD, CNRS UMR6293, INSERM U1103, Faculty of Medicine, Bldg CRBC, 2nd floor, 28 place Henri Dunant, 63001 Clermont-Ferrand, France; 2 Université Clermont Auvergne, 63001 Clermont-Ferrand, France; 3 CHU Clermont-Ferrand, Biostatistics unit, DRCI, 63003 Clermont-Ferrand, France; 4 Université de Lyon, Université Claude Bernard Lyon 1, CNRS UMR 5310, INSERM U 1217, LabEx DEVweCAN, Institut NeuroMyoGène (INMG), Lyon, France; 5 Université de Lyon, Université Claude Bernard Lyon 1, INSERM 1052, CNRS 5286, Centre Léon Bérard, Centre de recherche en cancérologie de Lyon, Lyon, France; 6 Institut de Biopathologie moléculaire, UF de biologie moléculaire, Centre de Bio-Pathologie Est, Hospices Civils de Lyon, Lyon, France; 7 AP-HP, Groupe Hospitalier Pitié-Salpêtrière Charles Foix, Service de neurologie 2-Mazarin, 75013 Paris, France; 8 Sorbonne Université, INSERM, CNRS, UMR S1127, Institut du Cerveau et de la Moelle épinière, ICM, Paris, France; 9 AP-HP, Hôpitaux Universitaires La Pitié Salpêtrière - Charles Foix, Service de Neuropathologie-Escourolle, 75013 Paris, France; 10 Hospices Civils de Lyon, Hôpital Pierre Wertheimer, Service de Neuro-oncologie, Bron, France; 11 Department of Cancer Cell Plasticity, Cancer Research Centre of Lyon, INSERM U1052, CNRS UMR5286, Lyon, France; 12 APHM, Hôpital de la Timone, Service d’Anatomie Pathologique et de Neuropathologie, Marseille, France; 13 Aix-Marseille University, CNRS, INP, Institut Neurophysiopathologie, Marseille, France; 14 CIMB, CNRS UMR9187, INSERM U1196, Institut Curie, 15 rue Georges Clemenceau, 91405 Orsay, France; 15 Département de Radiothérapie, Centre Jean Perrin, Clermont-Ferrand, France; 16 Département de Radiothérapie, Institut Curie, Paris, France

**Keywords:** Anaplastic astrocytoma, Secondary glioblastoma, Alternative lengthening of telomeres, IDH1/2 mutations, ATRX loss of expression

## Abstract

All cancer cells need to maintain functional telomeres to sustain continuous cell division and proliferation. In human diffuse gliomas, functional telomeres are maintained due either to reactivation of telomerase expression, the main pathway in most cancer types, or to activation of a mechanism called the alternative lengthening of telomeres (ALT). The presence of IDH1/2 mutations (IDH-mutant) together with loss of ATRX expression (ATRX-lost) are frequently associated with ALT in diffuse gliomas. However, detection of ALT, and *a fortiori* its quantification, are rarely, if ever, measured in neuropathology laboratories. We measured the level of ALT activity using the previously described quantitative “C-circle” assay and analyzed it in a well characterized cohort of 104 IDH-mutant and ATRX-lost adult diffuse gliomas. We report that in IDH-mutant ATRX-lost anaplastic astrocytomas, the intensity of ALT was inversely correlated with age (*p* < 0.001), the younger the patient, the higher the intensity of ALT. Strikingly, glioblastomas having progressed from anaplastic astrocytomas did not exhibit this correlation. ALT activity level in the tumor did not depend on telomere length in healthy tissue cells from the same patient. In summary, we have uncovered the existence, in anaplastic astrocytomas but not in glioblastomas with the same IDH and ATRX mutations, of a correlation between patient age and the level of activity of ALT, a telomerase-independent pathway of telomere maintenance.

## Introduction

Telomeres, first defined as structures located at the distal extremities of linear chromosomes with a specific function in preventing fusions between chromosome ends, are composed of 10 to 15 kb of repeated TTAGGG sequences [20, 27, 28]. Telomeres naturally erode with consecutive cell divisions, due to intrinsic mechanisms associated with the fixed 5′ to 3′ polarity of DNA replication, the so-called “end replication problem” [36]. Normally, telomerase, a reverse transcriptase enzyme with a built-in RNA template, compensates for this natural loss of telomeric sequences. However, due to the natural inactivation of telomerase, mainly by transcriptional mechanisms, telomeres of most somatic human tissues progressively shorten over time. This provokes a DNA damage-induced cell cycle arrest, which is the equivalent of replicative senescence in cultured cells [20, 31].

Following cancer initiation, tumor cells must overcome the telomere-controlled replicative senescence barrier to be able to proliferate indefinitely. To do this, they need to reactivate a pathway of maintenance of functional telomeres to keep them at a minimal length compatible with minimal chromosome stability [7]. There are two major pathways of telomere maintenance mechanisms in tumor cells. One is reactivation of telomerase, *hTERT*, which occurs in ~ 85–95% of cancer types [16, 18], principally as the result, in diffuse gliomas, of the occurrence of mutations in *hTERT* promoter [23]. The second mechanism is the so-called ALT (alternative lengthening of telomeres) pathway [3]. ALT functions using recombination between the repeated telomeric DNA sequences on two different telomeres, or between a telomere and extra-chromosomal telomeric circles of DNA, or, else, by sister chromatid exchange of telomeric DNA. There is also good evidence that a sort of “rolling circle” mechanism could also lead to telomere amplification on a single telomere [33].

Human diffuse gliomas are among the ~ 5–15% of cancer types that can survive either owing to telomerase or the ALT pathway [16, 18]. The ALT pathway is prevalent in some glioma subtypes and strongly associated with astrocytomas and secondary glioblastomas (GBM). Clinical studies indicate that, all grades and subtypes considered, ~ 30% of gliomas develop the ALT pathway. In recent years, the molecular landscape of gliomas has been intensively studied [4, 37]. In diffuse gliomas, ALT activation has been associated with mutations in IDH1 or IDH2 and ATRX [32]. There are two main groups of IDH1/2 mutant gliomas: (i) astrocytomas exhibiting ATRX mutation, TP53 mutation and ALT activation, (ii) oligodendrogliomas harboring 1p/19q codeletion and *hTERT* promoter mutation with *hTERT* overexpression.

Better understanding the ALT pathway of telomere maintenance has now become a stimulating challenge, as recent research aimed at developing therapeutic approaches targeting ALT [8, 22]. For instance, ALT tumors were recently found to be more sensitive than telomerase positive tumors to inactivation of the ATR kinase [12]. Two highly potent and selective ATR inhibitors are now being tested in clinical trials [22]. In addition, a ligand to G-quadruplex DNA has recently been shown to specifically inhibit the ALT pathway in glioma stem cells [21]. It is unknown yet why ALT is more frequent in some cancer subtypes and how ATRX, a recently demonstrated inhibitor of ALT, acts [6, 29]. There are currently several techniques for measuring the occurrence of the ALT pathway. Detection of ALT-associated promyelocytic leukemia (PML) nuclear bodies (APB) at the telomeres is a technique combining anti-PML immunofluorescence and telomere FISH [38]. A variation of the APB assay now uses telomere FISH to detect ultra-bright telomeric signals corresponding to the very long ALT telomeres [15, 16]. In addition, ALT is associated with the production of partially single-stranded extrachromosomal telomeric DNAs highly specific for ALT, the C-circles [17]. Application of a C-circle assay that amplifies the C-circles present in tumor DNA marked a major improvement in the detection of ALT, because it is highly specific, sensitive and quantifiable, and requires as little as 30 ng of DNA [17].

The main objective of the present study was to know whether quantifying ALT activity in diffuse glioma tumors could be informative in terms of basic and clinical interest. Interestingly, we observed an inverse correlation between patient age and ALT intensity in IDH1/2-, ATRX-mutated anaplastic astrocytomas, ALT intensity being significantly higher in younger patients. Strikingly, this patient age/ALT intensity correlation was not observed in secondary GBM with the same IDH and ATRX mutations. This is particularly interesting as these secondary GBM are thought to derive from anaplastic astrocytomas.

## Materials and methods

### Patients cohort from the French POLA network

One hundred and four patients from the French nation-wide POLA network were included in this study. Inclusion criteria were the written consent of the patient for clinical data collection and genetic analysis according to the national and POLA network policies, an established diagnosis of high-grade gliomas (grade III or IV) according to the WHO 2016 classification of nervous system tumors [25, 35], availability of clinical data collection (gender, age at surgery, tumor location, contrast-enhancement on MRI, extent of surgical resection, post-operative treatment and outcome) and sufficient material for molecular studies available. The study was approved by the ethics committee of Hôpital Universitaire La Pitié-Salpêtrière.

Each sample was analyzed under a light microscope after hematoxylin-eosin staining for assessing necrosis and tumor cell percentage. Samples with less than 70% of tumor cells were excluded from this study. Automated immunohistochemistry (IHC) was performed for IDH1 R132H neomorphic enzyme, ATRX nuclear expression, Ki67 and p53 as previously described [11]. When IDH1 R132H IHC was negative or unreliable, IDH1 and IDH2 mutational status was evaluated by direct sequencing using the Sanger method as previously described [35]. The genomic profile and assessment of the 1p/19q codeletion status was determined based on SNP arrays, CGH arrays, or microsatellite marker analysis as previously described [19]. *hTERT* promoter mutation status was evaluated by Sanger sequencing as previously described [24]. Patient age was considered at the time of first surgery.

### Measurements of ALT-specific C-circles

**Fig. 1:**
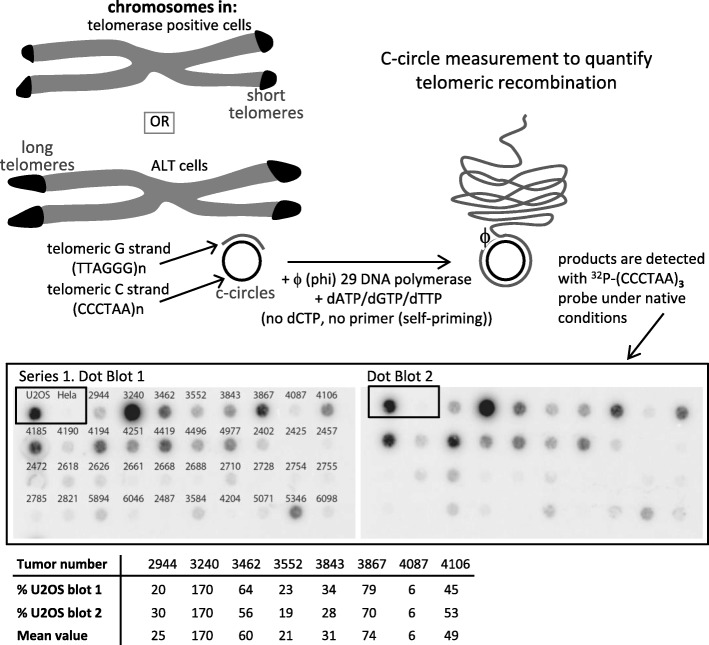
Measurement of the level of ALT activity levels in human diffuse gliomas. Top panels illustrate the principle of the ALT C-circle assay [17] and describe its general steps. ALT cells have very long telomeres that have been amplified mainly by homologous recombination that generates partially single-stranded extra-chromosomal circles. Genomic DNA prepared from tumor samples is then incubated with the Phi29 DNA polymerase that specifically amplifies this telomeric DNA. Middle panel illustrates ALT-specific signals measured in tumor DNA samples using this assay, which were detected here on dot blots hybridized with a telomeric ^32^P-labeled probe. Genomic DNAs from HeLa (telomerase positive) and U2OS (ALT positive) cells were also probed, representing negative and posititve controls for the C-circle assay, respectively. These assays were systematically performed in duplicates and here dot blot 2, on the right, was loaded with the same tumor samples as dot blot 1, on the left, to insure for reproducibility. Bottom table illustrates examples of duplicate numbers obtained for each of the indicated tumors, real signals of which are represented in the middle panel above. The C-circle score was determined after calculating the intensity of the signal relative to that of the ALT positive U2OS cell line, designated to be 100 arbitrary units (AU). Note that the C-circle assays were performed on representative samples, including those from the two patient groups analyzed in the present study

The C-circle (CC) assay detects partially single-stranded telomeric (CCCTAA)n, ALT-specific, DNA circles (C-circles) following amplification by the Phi29 polymerase in the absence of dCTP [17]. The previously described C-circle assay [17], used here to quantify ALT activity, has already been used in our laboratory [9, 13]. Figure 1 depicts this assay in its general outlines. Genomic DNA, prepared from fresh frozen tissue, was digested with 4 U/μg *Hinf*I and *Rsa*I restriction enzymes and 25 ng/μg of Dnase-free RNase. Ten μl of each sample (30 ng) was combined with 10 μl 0.2 mg/ml BSA, 0.1% Tween, 1 mM each dATP, dGTP and dTTP, 1X Phi29 Buffer and 7.5 U Phi29 DNA polymerase (Thermo Scientific, Fermentas) and incubated at 30 °C for 8 h, then at 65 °C for 20 min. The reaction products were diluted to 60 μl with 2X SSC and dot-blotted onto a 2x SSC-soaked Hybond N^+^ nylon membrane (GE Heathcare). DNA was UV-cross-linked onto the membrane, which was then hybridized at 37 °C with end-labeled ^32^P-(CCCTAA)_3_ and PerfectHyb Plus hybridization buffer (Sigma Aldrich). Figure 1 also illustrates ALT-specific signals measured in tumor DNA samples. In addition to the tumor samples, each experiment contained both positive and negative control genomic DNAs from two cell lines, U2OS and HeLa, which are the prototypes of ALT positive and telomerase positive cells, respectively. Following measurement of the intensity of each spot (one spot representing one tumor), the C-circle value of each tumor was calculated relative to that measured in genomic DNA from the ALT positive U2OS cells, designated to be 100 arbitrary units (AU). All measurements were performed in duplicates. Results were analyzed using a GE Storm phosphorimager and quantified using ImageQuant software.

### Detection of ALT by visualization of ultra-bright telomeric signals (Telo-FISH)

In the fluorescence microscope, promyelocytic leukemia nuclear bodies (PML) signals, detected with anti-PML antibody, are usually identified as corresponding to APB (ALT-associated PML bodies) when they co-localize with the FISH-labeled telomeres [18, 38]. However, recently, Heaphy et al. [15] found cases of fixed tumoral tissues in which ultra-bright Telo-FISH signals did not co-stain with PML. Since cultured cells lacking co-localization between Telo-FISH signals and PML, but otherwise shown to be ALT positive, had been described [5, 10, 26], Heaphy et al. [15], next followed by several different laboratories, adopted detection of ultra-bright Telo-FISH signals as the most accurate marker of ALT in fixed tissue specimens.

Paraffin sections (6 μm thickness) were deparaffinized and dehydrated in an ethanol series. Sections were air-dried before in situ hybridization at 80 °C for precisely 3 min with the telo-PNA (peptide nucleic acid) probe (Cy3-OO CCC TAA CCC TAA CCC TAA; 0.5 ng; Applied Biosystems, Foster City, CA, USA) dissolved at 5 μg/mL in a hybridization mix containing 70% formamide, 10 mM Tris (pH 7.2), 5% Mg-buffer (25 mM MgCl_2_, 9 mM citric acid, and 82 mM Na_2_HPO_4_), and 0.5% Boehringer blocking powder. Following 1 h incubation in the dark at room temperature, slides were washed twice for 15 min in 50% formamide, 10 mM Tris (pH 7.2), and 0.1% BSA, then three times for 5 min each in 100 mM Tris (pH 7.5), 100 mM NaCl, and 0.08% Tween-20. Sections were washed and counterstained with 3 μg 4′,6-diamidino 2-phenylindole (DAPI). Images were captured on a Zeiss Axioimager Upright Microscope, using 40X magnification, and analyzed with Columbus Image Data Storage and Analysis System (PerkinElmer). Nuclei were delineated using DAPI counterstaining, and spot number and area were automatically calculated (over 900 nuclei per patient). Sections were scored as positive for ultra-bright telomeres, if at least 6 telomeric foci were detected per nucleus and over 45% of the foci were larger than 20 pixels.

### Telomere length measurement by telomere restriction fragment (TRF) analysis

Measurement of telomere length was performed by Southern blot analysis, also referred to as TRF (Telomere Restriction Fragment) analysis as previously described [2]. Briefly, 5 μg of genomic DNAs extracted from patients blood were digested with *Rsa*I *and Hinf*I and separated in a 0.9% agarose gel (in TBE) run in TBE buffer overnight and, after denaturation, transferred and hybridized with a (TTAGGG)_3_^32^P-labeled telomeric probe. Following digestion of genomic DNA, telomere tracts appear as a broad band which represents the average length of most telomeres. This smear is very heterogeneous because telomere length not only varies between chromosome ends, but also between cells. Results were analyzed using a GE Storm phosphorimager and quantified using ImageGauge software.

### Statistical analysis

All analyses were performed using Stata software (Version 13, StataCorp, College Station, TX, USA) for a two-sided type I error of α = 5%. Patients’ characteristics were expressed as mean ± standard deviation (SD) or median (interquartile range) for continuous data (assumption of normality assessed by using the Shapiro-Wilk test) and as numbers and associated percentages for categorical parameters. Correlation coefficients (Pearson or Spearman according to statistical distribution) were estimated to study relationships between quantitative parameters. As the statistical distribution of ALT activity was not Gaussian (for all patients and for each subgroup), the correlation coefficient estimated to study the relationships between ALT activity and other parameters was non parametric (Spearman). Quantitative variables were then compared between groups using Student t-test or non-parametric Mann-Whitney test if t-test assumptions were not met (normality and homoscedasticity analyzed using the Fisher-Snedecor test). For categorical parameters, the comparisons between groups were carried out with Chi-squared or Fischer’s exact tests. Overall survival and disease free survival were studied as a censored data. Therefore, comparisons were analyzed using log-rank test and Cox proportional-hazards regression. The proportional-hazard hypothesis was studied using Schoenfeld’s test and plotting residuals. Results were expressed as hazard ratios (HR) and 95% confidence intervals.

## Results

Definition of diffuse glioma patients groups

Based on histomolecular characteristics described in the Materials and methods section, we selected, according to the WHO 2016 classification of brain tumors, 60 IDH-mutant anaplastic astrocytomas (AA) and 44 IDH-mutant GBM that all exhibited loss of ATRX. The percentages of p53 immunoreactivity and Ki67 positive tumor cells were also assessed. Measurements of these molecular markers in all 104 patients, plus the C-circle score (see below) and the presence of mutations in *hTERT* promoter, as well as all available clinical parameters are shown in Additional file 1: Table S1.

### The level of ALT activity correlates with patient age in IDH-mutant ATRX-lost AA

**Fig. 2:**
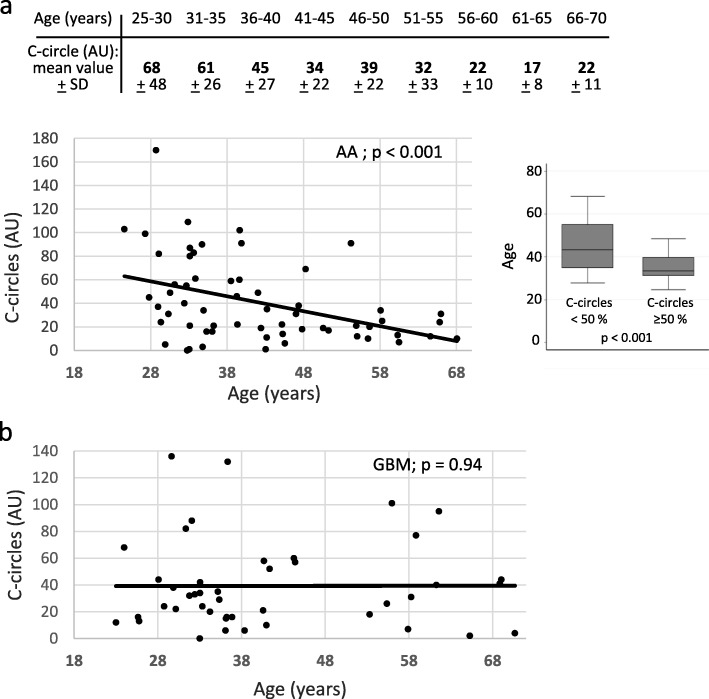
Correlation between the level of ALT activity, expressed as the percentage of radioactive signal with respect to the ALT signal recorded in genomic DNA from U2OS cells, designated to be 100 arbitrary units (AU), and patient age in anaplastic astrocytomas, AA (**a**, top and bottom left) and secondary glioblastomas multiform, GBM (**b**). Patient age was considered at the time of first surgery. **a** bottom right: According to statistical distribution, we arbitrarily defined two categories of anaplastic astrocytoma (AA) patients with C-circle score < or > 50 AU. This graphical representation also clearly shows the ALT intensity/patient age correlation

Compared with telomerase positive cells, ALT cells have very long telomeres (up to 50 kb) that are generated by recombination-dependent DNA replication [33]. ALT cells, but not telomerase positive cells, generate extra-chromosomal telomeric single-stranded DNA called C-circles, which can be amplified using the Phi29 DNA polymerase [17] (Fig. 1). C-circle scores for all analyzed IDH-mutant ATRX-lost tumors, together with other molecular markers are shown in Table 1. In the AA population, we found a significant inverse correlation (− 0.41, *p* < 0.001) between the level of ALT activity and patient age, meaning that the younger the patient, the higher the ALT-associated C-circle value (Fig. 2a, bottom). In this group, ALT positive patients with ages comprised between 25 and 30 years had tumors with a mean C-circle intensity of around 68 AU (arbitrary units; see Materials and methods), while ALT positive 66–70 years-old patients had tumors with a mean C-circle intensity of around 22 AU, all 5-year-classes in-between having intermediate, age proportional increasing values of C-circles (Fig. 2a, top). It was important to know whether the C-circle score/age correlation observed in the ALT positive AA also existed in the GBM with the same IDH-mutant ATRX-lost genotype. Interestingly, in the 44 analyzed GBM, there was no significant correlation between the level of ALT activity and patient age at the time of surgery (correlation of 0.01, *p* = 0.94; Fig. 2b). We note that, for these experiments, the correlation coefficient was very weak, close to zero, which led us to conclude that the lack of significant age/ALT intensity correlation in the GBM was not due to a lower statistical power analysis compared with the AA. When the entire cohort was considered (*n* = 104), a weak correlation between age and ALT activity level was found (correlation of − 0.23, *p* = 0.02).

**Table 1:** Clinical parameters and molecular analyses of the tumors

Tumor number anaplastic astrocytomas	p53	Ki67	age at first symptoms	TERT promoter	C-circles (arbitrary units)
3570	5%	7%	56	wt	20
4080	50%	10%	56	wt	6
4140	50%	70%	28	wt	82
4827	60%	5%	32	wt	55
5595	30%	12%	54	wt	91
6637	80%	10%	33	wt	87
4185	40%	15%	33	wt	80
3552	70%	10%	32	wt	21
4644	60%	5%	66	wt	31
3635	3%	15%	43	wt	19
3843	90%	10%	29	wt	31
3091	80%	7%	68	wt	10
4126	50%	15%	61	wt	7
4975	50%	7%	55	wt	10
3240	5%	15%	NA	wt	170
3402	negative	3%	38	wt	1
4031	50%	10%	47	wt	31
4878	negative	15%	24	wt	103
5815	negative	12%	45	wt	14
3462	60%	10%	40	wt	60
4951	60%	10%	43	wt	11
5261	60%	5%	38	wt	59
5489	10%	30%	48	C250T	69
5546	80%	10%	27	wt	99
5841	60%	10%	58	wt	34
5895	100%	5%	51	wt	19
6013	60%	6%	33	wt	16
6095	negative	8%	NA	wt	56
6658	60%	12%	33	wt	3
3558	100%	20%	39	wt	22
4056	50%	15%	29	wt	5
4289	90%	10%	28	wt	24
4572	90%	8%	42	wt	49
4840	75%	8%	34	wt	61
5238	80%	8%	55	wt	21
5744	50%	12%	34	wt	83
5987	80%	15%	35	wt	90
5443	80%	12%	61	wt	12
5448	50%	12%	29	C228T	37
5790	50%	12%	47	wt	18
5793	60%	15%	43	wt	35
5866	60%	15%	40	wt	91
6244	60%	20%	45	wt	22
6309	60%	12%	39	wt	102
4569	90%	15%	32	C250T	40
2944	70%	10%	57	wt	25
5022	negative	12%	66	wt	24
5725	80%	12%	53	wt	12
6011	80%	20%	39	wt	46
5974	80%	5%	34	wt	16
5003	30%	5%	33	C228T	109
3684	25%	7%	33	C228T	1
4106	negative	10%	31	wt	49
5593	60%	10%	34	wt	34
5594	80%	11%	59	wt	13
6654	60%	8%	52	wt	17
6742	40%	4%	48	wt	38
5510	35%	20%	28	C228T	45
5912	80%	12%	33	wt	0
5398	80%	12%	36	wt	21
Tumor number secondary GBM	p53	Ki67	patient age (years)	TERT promoter	C-circles
3942	80%	25%	37	wt	6
2592	40%	10%	69	wt	41
3077	60%	20%	25	wt	16
3007	80%	30%	31	wt	82
3297	30%	70%	59	wt	77
4034	30%	10%	58	wt	7
4115	80%	25%	55	wt	101
3845	80%	8%	32	wt	32
3928	negative	30%	41	wt	58
3933	negative	25%	32	wt	88
3992	40%	15%	34	wt	20
3959	90%	15%	26	wt	13
3019	100%	25%	29	wt	136
2835	20%	8%	37	wt	52
3316	40%	70%	24	wt	68
2695	60%	60%	30	wt	38
3359	100%	60%	41	wt	10
3451	40%	3%	61	wt	40
4068	60%	20%	NA	wt	44
4082	negative	12%	35	wt	35
4125	60%	25%	36	wt	6
4145	30%	12%	29	wt	29
4194	70%	10%	33	wt	34
4245	15%	12%	29	wt	24
4234	80%	20%	30	wt	22
4554	60%	10%	33	wt	24
4602	60%	12%	65	C250T	2
4711	20%	10%	57	wt	31
4706	80%	40%	39	wt	132
4707	90%	30%	40	wt	21
5007	60%	12%	53	wt	18
5014	100%	30%	62	wt	95
4918	60%	15%	36	wt	15
5067	95%	15%	33	wt	42
5246	80%	5%	36	wt	16
5248	80%	30%	44	wt	60
5160	60%	12%	67	wt	44
5137	50%	50%	32	wt	33
5152	95%	12%	72	wt	4
5169	60%	20%	36	C228T	16
5543	80%	10%	23	wt	12
5876	40%	10%	32	wt	0
5839	5%	12%	55	wt	26
5983	negative	25%	43	wt	57

In the two groups of patients, similar mean age values were present within the different quartiles, thus showing that the ALT intensity/age correlation found in AA was not biased by age distribution (Additional file 2: Table S2). In addition, the ALT intensity/age correlation found in AA was not biased by potentially large differences in the mean C-circle values between the two groups, because these were very similar, 40.95 + 34.96 AU for AA vs 39.25 + 33.05 AU for GBM.

### Measurements of ALT activity in patients with relapse having evolved from lower grade or anaplastic astrocytoma to GBM

Given that the secondary GBM tumors are thought to derive from either low-grade or anaplastic astrocytomas, it was particularly remarkable that the patient age/ALT intensity correlation was observed in AA but not in secondary GBM. To better understand these mechanisms, we measured C-circle activity in matched samples from patients having evolved from astrocytoma to GBM. We identified 13 paired cases, among which only eight could be exploited, due to the fact that DNA extracted from paraffin-included tumors did not give signals in the C-circle assay under the conditions used for frozen tumor samples (data not shown). All relapsed tumors remained IDH-mutant, ATRX-lost and 1p/19q non-codeleted. In these experiments, we noted that all four patients with high ALT intensity (> 60 AU) nevertheless evolved to GBM (Additional file 3: Table S3). This suggested that an initial high level of C-circle in these four young patients (22 to 40 years old) did not prevent progression from AA to GBM. In some, but not all, patients, C-circle intensity remained rather low or rather high in GBM with respect to the first measurement in astrocytomas (Additional file 3: Table S3). Although these data are somewhat informative, they cannot elucidate the problem of knowing why AA and GBM differ in terms of age/ALT intensity correlation, which might nevertheless be possible if performed on a much larger number of patients with relapse.

### Telomere length in somatic cells does not correlate with ALT intensity in tumors

Telomeres of human somatic cells erode with age. One might hypothesize that telomeres of somatic cells of younger patients are longer than those of older patients, which could be the reason why the ALT pathway is more intense in these patients. Therefore, we next set out to measure telomere length in peripheral blood mononuclear cells of patients chosen from several distinct classes of age and ALT characteristics (Additional file 4: Figure S1). These experiments clearly established that telomere length in healthy cells at the time of oncogenic transformation is not correlated with the intensity of the ALT mechanisms in the emerging tumor. Thus, for instance, among young AA patients, there was no difference in telomere length in mononuclear cells whether the tumors exhibited low, medium or high C-circle level. This was the same for older AA patients. In addition, there was no difference in telomere length of peripheral blood cells between AA and oligodendrogliomas patients (Additional file 4: Figure S1).

### Correlations between the amount of ALT-specific C-circles and the presence of ALT-associated ultra-bright telomeric foci

**Fig. 3:**
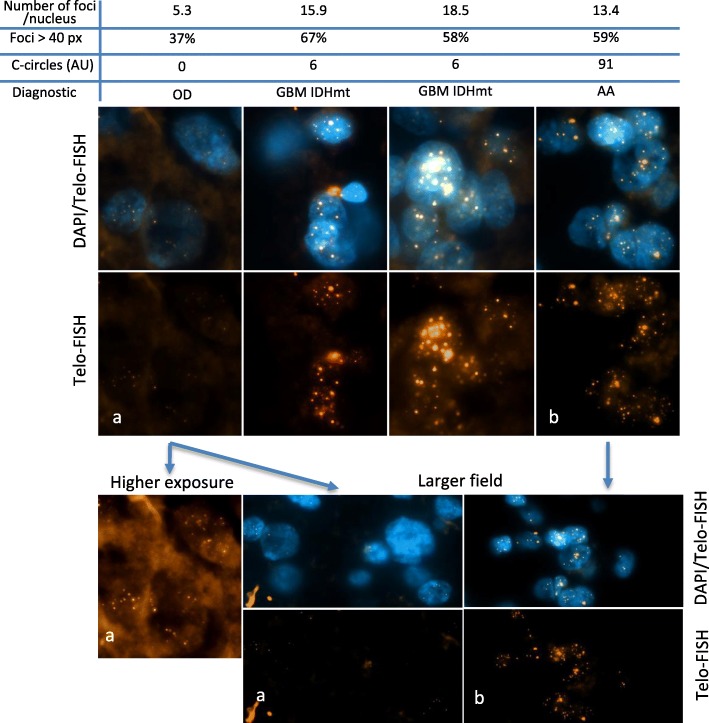
Detection of ultra-bright telomeric foci by Telo-FISH in paraffin-embedded sections of tumors, together with detection of DNA by DAPI in the same section, as indicated (40X magnification). The upper chart indicates the mean number of telomeric foci per nucleus, as well as the percentage of nuclei with ultra-bright foci of an intensity over 40 pixels (px), the rate of C-circles and the diagnostic. C-circles values are expressed in arbitrary units (AU; see Materials and methods). The images are representative pictures of tumors; OD: oligodendroglioma; GBM IDHmt: IDH-mutant secondary glioblastoma; AA: anaplastic astrocytoma. Note that the photograph representing the OD tumor (picture a) was taken at a much higher exposure, as indicated, to show that the Telo-FISH signals are indeed present, but are much fainter than in the GBM and AA tumors. For both the OD tumor (**a**) and AA tumor (**b**) shown in the upper panels, lower magnifications are provided in the lower panels, as indicated (larger field)

In contrast to high score ALT tumors, for instance tumor #3240 (170 AU), a substantial number of tumors had a much lower score (Fig. 1; Table 1). An important issue was to determine whether these low to very low C-circle tumors developed true ALT mechanisms. To achieve this, we set out to visualize the presence of ultra-bright telomeric foci by Telo-FISH. Detection of these foci in the fluorescence microscope is now widely used to identify the very long and heterogeneous telomeres associated with ALT (see Materials and methods). Using this approach, we found that all nine fixed paraffin-embedded tumors in which ALT-specific C-circles had been detected, in the corresponding frozen tissue, with a score equal or greater than 6 AU also contained ALT-specific ultra-bright Telo-FISH foci (Fig. 3). Two additional tumors with a C-circle score of 2 or 4 AU also contained ALT-specific ultra-bright Telo-FISH foci, but these foci were found to be smaller than in the other nine tumors examined (the tumor with the 2 AU score was in fact *hTERT*-mutated; data not shown). On the other hand, five oligodendroglioma tumors (with functional ATRX and a mutation in *hTERT*), measured with a C-circle score of 0 AU, did not exhibit ALT-specific ultra-bright Telo-FISH foci (Fig. 3). Based on these experiments, we conclude that a threshold of C-circle value of 6 AU defines with confidence tumors that have ALT activity. These conclusions are reinforced by the finding that in 30 oligodendrogliomas that were 1p/19q codeleted, ATRX-wt, *hTERT*-mutated and IDH-mutant, the average C-circle score was 0.8 AU (data not shown).

### High Ki67 levels correlate with ALT activity level in GBM

We also paid particular attention to p53 immunoreactivity and Ki67 levels. The correlation coefficient between p53 score and C-circle intensity was − 0.11 (*p* = 0.41) for the 60 analyzed AA and − 0.10 (*p* = 0.53) for the 44 analyzed GBM. On the other hand, interestingly, we found a moderate correlation between Ki67 levels and C-circle intensity in GBM (0.38, *p* = 0.01), but not in AA (correlation coefficient of 0.20, *p* = 0.12). However, the fact that Ki67 values were clearly lower in AA than in GBM (mean of 12.13 + 8.96 for AA vs 22.27 + 16.63 for GBM) prevented us from drawing conclusions concerning the presence or absence of correlation between Ki67 and C-circle intensity in AA (Additional file 5: Table S4).

### Absence of relationships between survival and ALT activity level

There was no statistically significant correlation between ALT intensity (C-circle score) or patient age and overall survival both in AA and GBM patients (Additional file 6: Table S5). We also note that there was no significant difference in overall survival between AA and GBM patients (Additional file 6: Table S5). Therefore, the ALT intensity/patient age correlation we observe in the AA group does not result from the fact that younger patients have a higher level of ALT activity because they survive longer than older patients.

## Discussion

The ALT pathway represents an alternative to telomerase in maintaining functional telomeres, which is essential for cell division and proliferation of tumor cells. ALT is prevalent in IDH-mutant ATRX-lost diffuse gliomas and we now propose, based on the present data, that its detection and quantification in pathology laboratories might be very useful. The major finding of the present study is that in IDH-mutant ATRX-lost anaplastic astrocytomas (AA), the younger the patient with ALT positive tumor, the stronger the ALT-associated C-circle signal. IDH-mutant ATRX-lost GBM, with similar age distribution, failed to exhibit this correlation. Incidentally, we note that it was previously shown that in astrocytomas, GBM and osteosarcomas, ALT positive patients were younger than non-ALT patients [14, 18]. Most importantly, the ALT/age correlation we have found here concerns the intensity of ALT, not only its occurrence, contrary to these previous studies that examined ALT occurrence, but not its level of activity.

ATRX functions as an inhibitor of ALT in cancer cell lines [6, 29]. ATRX also seems to function as an inhibitor of ALT in vivo, as mutations in ATRX are associated with –and define- the ALT pathway in tumors. In addition, the few tumors of this study that exhibited both a mutation in *hTERT* promoter and ATRX were clearly ALT positive (Table 1), thus pointing out to ALT being epistatic to telomerase gain of function. The concomitant presence of these two mutations is rather unusual, but is nevertheless known to occur. Given the reliability and reproducibility of the C-circle assay as a molecular marker of ALT [17], the finding that most of these tumors (5/6 for AA, 1/2 for GBM) have a significant level of ALT actitivity should be kept in mind for further investigation.

It is not known yet why the intensity of ALT can largely differ from one tumor to the next, even within the same group. This could be due to the fact that C-circle activity might be highly dynamic and be affected by activation/deactivation of various pathways. Alternatively, different types of ALT mechanisms may exist within the same group of tumors. Our measurements of ALT intensity in AA patients with relapse and progression to GBM [30] seem to rule out the existence of an AA-specific type of ALT, with high intensity, no longer present in GBM. Differences in ALT intensities in a given patient at different times and tumor stages, noted in these patients with relapse (Table S3), are potentially meaningful and would deserve to be further examined in much larger numbers of patients. Technically, such an approach could be greatly facilitated by measuring C-circle levels in patient blood [17].

Telomere length in humans varies with time, progressively shortening as people age (see, for instance, reference [1]). Long telomeres might be more prone to ALT-based recombination than shorter telomeres. Excessively short telomeres might also be more prone to ALT-based recombination, which could then be used as a DNA repair pathway. We found no correlation between the level of ALT activity in the tumor and telomere length in the corresponding normal tissue. Therefore, it is unlikely that the intensity of the ALT pathway in the emerging tumor is dependent on telomere length of the healthy tissue that underwent oncogenic transformation.

The significance of the level of ALT activity as a clinical parameter was demonstrated in the present study by monitoring, in complement to C-circles, for the presence of ALT-specific ultra-bright Telo-FISH signals. All nine tumors in which a C-circle score > 6 AU was detected indeed exhibited ultra-bright Telo-FISH signals, thereby demonstrating that these low-C-circle score tumors are true ALT positive tumors.

An additional technical issue stems from the fact that our C-circle measurements were performed on a single sample from each tumor (albeit in duplicates) and, consequently, did not take into account intra-tumor heterogeneity. In future studies, at least some of the measurements of ALT intensity should ideally be performed on several samples from the same tumor.

Ki67 measurement represents a reliable cellular marker for cell proliferation [34]. As expected, we found that in GBM, Ki67 staining (higher than in AA) was correlated with ALT intensity. However, we note that in young AA patients a low Ki67 staining could co-exist with high ALT activity, thereby suggesting that the level of ALT activity in AA is unrelated to cell proliferation.

## Conclusions

In summary, the present work demonstrates the importance and potential clinical significance of not only detecting, but also quantifying the ALT pathway, a telomere maintenance mechanism that functions when telomerase cannot be re-activated in the tumor. Using the C-circle assay, which requires only 30 ng of tumor DNA, we have shown that ALT intensity correlates with patient age in IDH1/2-, ATRX-mutated anaplastic astrocytomas (AA). This finding could certainly be used in a near future to delineate possible subtypes of ALT-positive AA based on the intensity of the ALT pathway by C-circle. On the other hand, it would be important to understand the correlation between ALT intensity and patient age at the molecular level. To do this, cells from patients with different ALT intensities could be derived for further molecular and genetic studies. The potential existence of different types of ALT would certainly open wide avenues of research, as well as design of targeted treatments, on the telomeric mechanisms that govern cell proliferation in cancer cells.

## Supplementary information

**Additional file 1: Table S1.** Clinical parameters and molecular analyses of the tumors.

**Additional file 2: Table S2.** Distribution of age within anaplastic astrocytomas (AA) and secondary glioblastoma (GBM) patients.

**Additional file 3: Table S3.** Measurement of ALT activity in patients with relapse.

**Additional file 4: Figure S1.** Telomere length in peripheral blood mononuclear cells from 25 patients using TRF analysis (5 μg DNA per sample).

**Additional file 5: Table S4.** Ki67/C-circle correlation in GBM.

**Additional file 6: Table S5.** Patients survival data.

## Data Availability

The datasets used and/or analyzed during the current study are available from the corresponding author on reasonable request.

## Abbreviations

AA: Anaplastic astrocytoma
ALT: Alternative lengthening of telomeres
APB: ALT-associated promyelocytic leukemia bodies
ATRX: α-thalassia/mental/retardation X-linked
AU: Arbitrary units
GBM: Glioblastoma multiform IDH Isocitrate dehydrogenase
IHC: Immunohistochemistry
MRI: Magnetic Resonance Imaging
TRF: Telomere restiction fragments
