# Supplementary material for: The level of activity of the alternative lengthening of telomeres correlates with patient age in IDH-mutant ATRX-loss-of-expression anaplastic astrocytomas
Source: Acta Neuropathol Commun. 2019 Nov 9;7:175. doi: 10.1186/s40478-019-0833-0 (PMC6842523; doi:10.1186/s40478-019-0833-0)
Supplement: Supplementary file 2 — Additional file 2: Table S2. Distribution of age within anaplastic astrocytomas (AA) and secondary glioblastoma (GBM) patients. [file 40478_2019_833_MOESM2_ESM.pptx]

## Slide 1
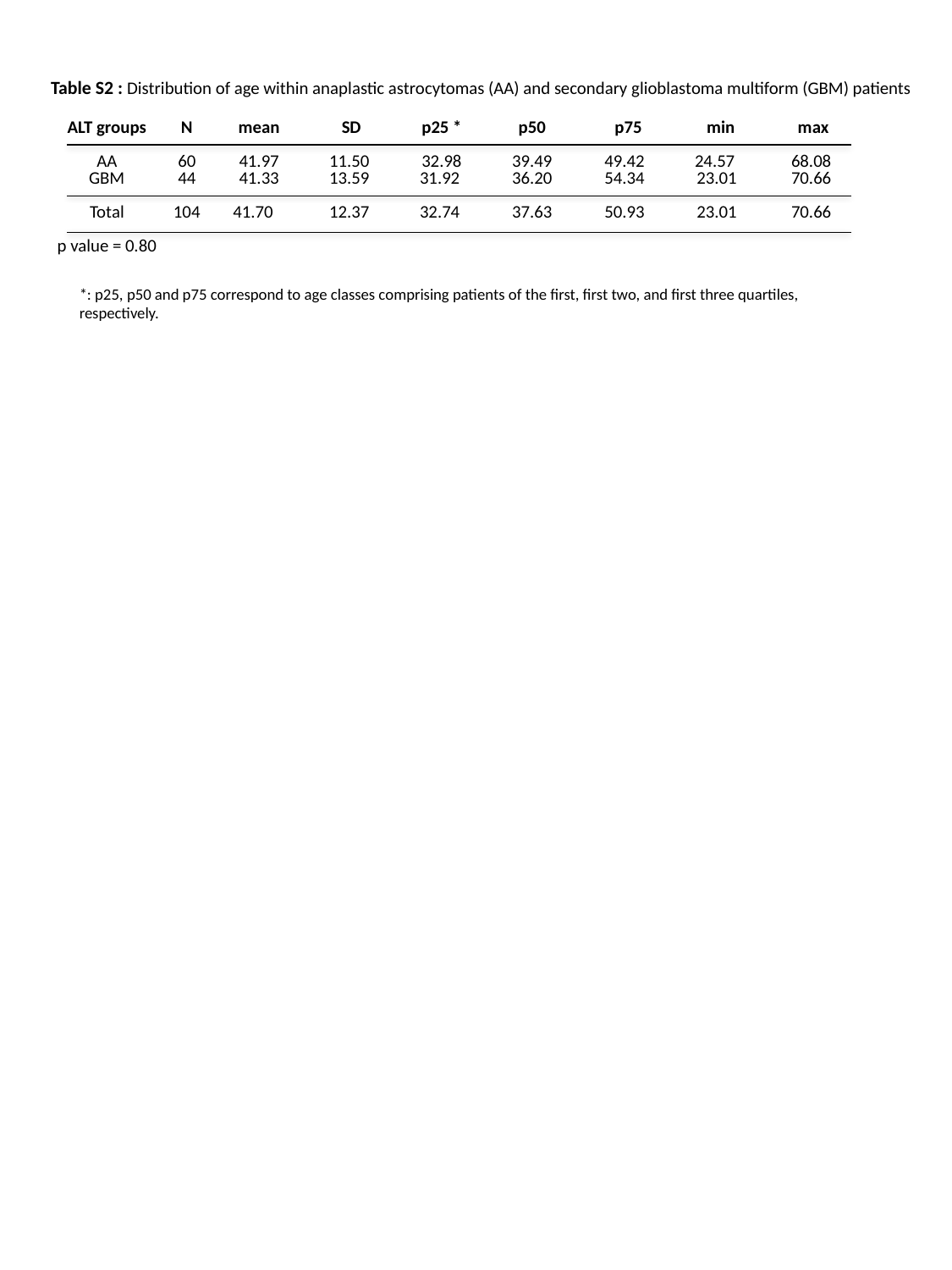

Table S2 : Distribution of age within anaplastic astrocytomas (AA) and secondary glioblastoma multiform (GBM) patients
ALT groups
AA
GBM
Total
p value = 0.80
N
60
44
104
mean
41.97
41.33
41.70
SD
11.50
13.59
12.37
p25 *
32.98
31.92
32.74
p50
39.49
36.20
37.63
p75
49.42
54.34
50.93
min
24.57
23.01
23.01
max
68.08
70.66
70.66
*: p25, p50 and p75 correspond to age classes comprising patients of the first, first two, and first three quartiles, respectively.
