# Supplementary material for: The level of activity of the alternative lengthening of telomeres correlates with patient age in IDH-mutant ATRX-loss-of-expression anaplastic astrocytomas
Source: Acta Neuropathol Commun. 2019 Nov 9;7:175. doi: 10.1186/s40478-019-0833-0 (PMC6842523; doi:10.1186/s40478-019-0833-0)
Supplement: Supplementary file 3 — Additional file 3: Table S3. Measurement of ALT activity in patients with relapse. [file 40478_2019_833_MOESM3_ESM.pptx]

## Slide 1
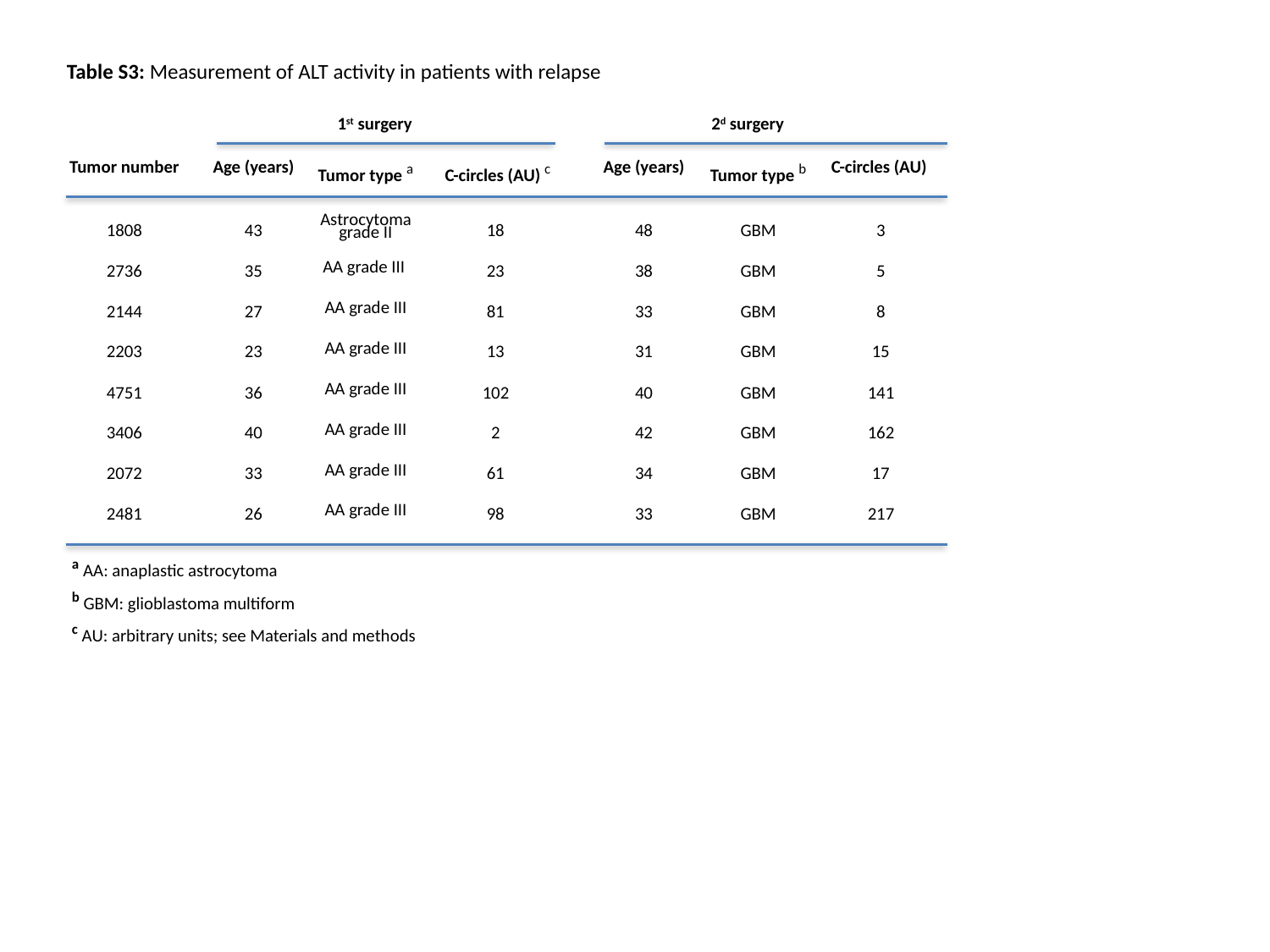

Table S3: Measurement of ALT activity in patients with relapse
1st surgery
2d surgery
Tumor number
Age (years)
Tumor type a
 C-circles (AU) c
Age (years)
Tumor type b
C-circles (AU)
Astrocytoma
grade II
1808
43
18
48
GBM
3
2736
35
23
38
GBM
5
AA grade III
2144
27
81
33
GBM
8
AA grade III
2203
23
13
31
GBM
15
AA grade III
4751
36
102
40
GBM
141
AA grade III
3406
40
2
42
GBM
162
AA grade III
2072
33
61
34
GBM
17
AA grade III
2481
26
98
33
GBM
217
AA grade III
a AA: anaplastic astrocytoma
b GBM: glioblastoma multiform
c AU: arbitrary units; see Materials and methods
