# Supplementary material for: The level of activity of the alternative lengthening of telomeres correlates with patient age in IDH-mutant ATRX-loss-of-expression anaplastic astrocytomas
Source: Acta Neuropathol Commun. 2019 Nov 9;7:175. doi: 10.1186/s40478-019-0833-0 (PMC6842523; doi:10.1186/s40478-019-0833-0)
Supplement: Supplementary file 4 — Additional file 4: Figure S1. Telomere length in peripheral blood mononuclear cells from 25 patients using TRF analysis (5 μg DNA per sample). [file 40478_2019_833_MOESM4_ESM.doc]

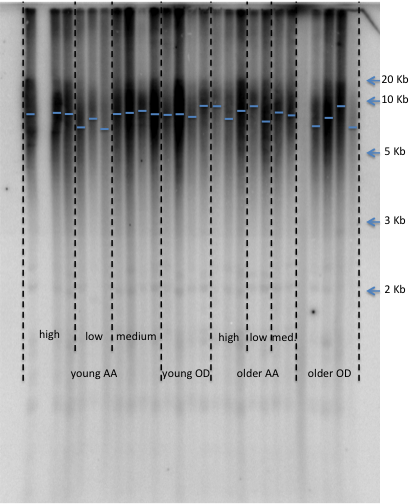


**Figure S1.** Telomere length was measured in peripheral blood mononuclear cells from 25 patients (one lane represents one patient) using TRF analysis (5 μg DNA per sample). Horizontal lines across each lane within the gel represent the calculated mean value of telomere length (the center of the smear). Note that the length of the smear is also important because it reflects the degree of scattering of all telomeres lengths around the mean value of the whole cell population. A ^32^P-labeled (TTAGGG)_3_ telomeric probe was used. OD: oligodendroglioma; AA: anaplastic astrocytomas. Low, medium and high refer to a C-circle value of 1-6 AU 16-37 AU and 58-102 AU, respectively.
