# Supplementary material for: The level of activity of the alternative lengthening of telomeres correlates with patient age in IDH-mutant ATRX-loss-of-expression anaplastic astrocytomas
Source: Acta Neuropathol Commun. 2019 Nov 9;7:175. doi: 10.1186/s40478-019-0833-0 (PMC6842523; doi:10.1186/s40478-019-0833-0)
Supplement: Supplementary file 5 — Additional file 5: Table S4. Ki67/C-circle correlation in GBM. [file 40478_2019_833_MOESM5_ESM.pptx]

## Slide 1
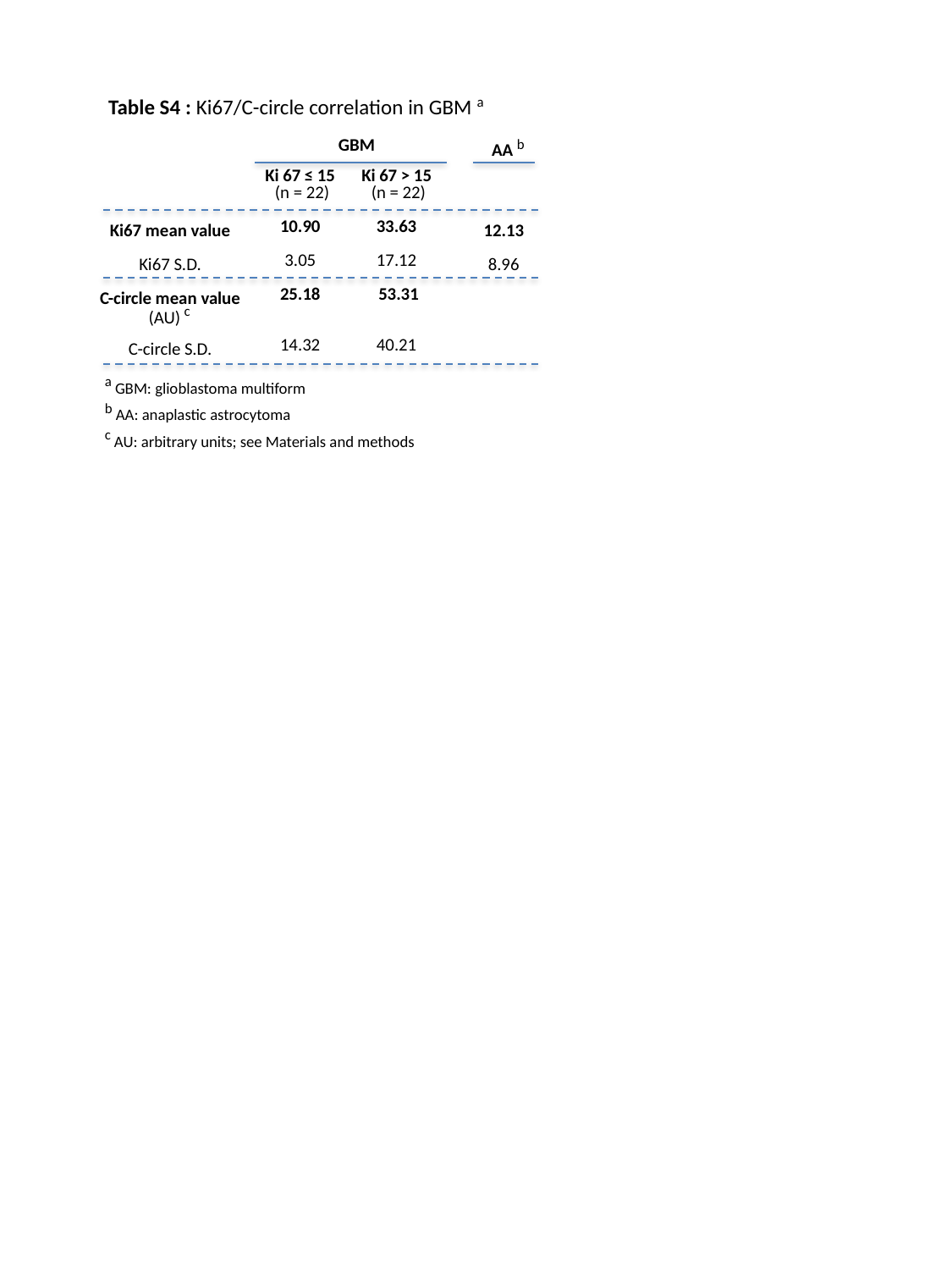

Table S4 : Ki67/C-circle correlation in GBM a
GBM
AA b
Ki67 mean value
Ki67 S.D.
C-circle mean value
C-circle S.D.
Ki 67 ≤ 15
(n = 22)
10.90
3.05
25.18
14.32
Ki 67 > 15
(n = 22)
33.63
17.12
53.31
40.21
12.13
8.96
(AU) c
a GBM: glioblastoma multiform
b AA: anaplastic astrocytoma
c AU: arbitrary units; see Materials and methods
