# Supplementary material for: The level of activity of the alternative lengthening of telomeres correlates with patient age in IDH-mutant ATRX-loss-of-expression anaplastic astrocytomas
Source: Acta Neuropathol Commun. 2019 Nov 9;7:175. doi: 10.1186/s40478-019-0833-0 (PMC6842523; doi:10.1186/s40478-019-0833-0)
Supplement: Supplementary file 6 — Additional file 6: Table S5. Patients survival data. [file 40478_2019_833_MOESM6_ESM.pptx]

## Slide 1
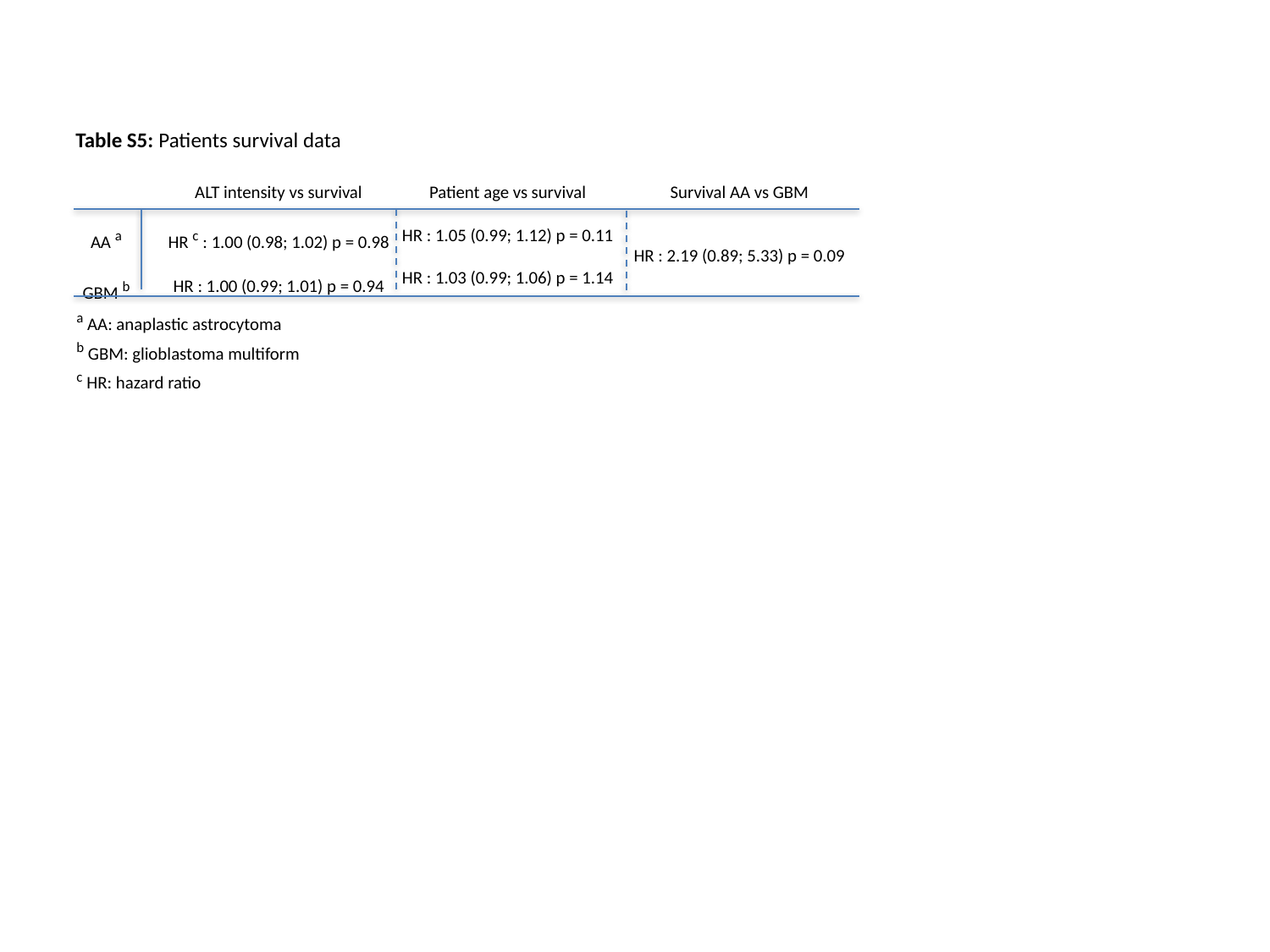

Table S5: Patients survival data
ALT intensity vs survival
HR c : 1.00 (0.98; 1.02) p = 0.98
HR : 1.00 (0.99; 1.01) p = 0.94
Patient age vs survival
HR : 1.05 (0.99; 1.12) p = 0.11
HR : 1.03 (0.99; 1.06) p = 1.14
Survival AA vs GBM
HR : 2.19 (0.89; 5.33) p = 0.09
AA a
GBM b
a AA: anaplastic astrocytoma
b GBM: glioblastoma multiform
c HR: hazard ratio
